# Supplementary material for: The interactome of intact mitochondria by cross-linking mass spectrometry provides evidence for coexisting respiratory supercomplexes
Source: Mol Cell Proteomics. 2017 Dec 8;17(2):216–32. doi: 10.1074/mcp.RA117.000470 (PMC5795388; doi:10.1074/mcp.RA117.000470)
Supplement: Supplemental Data [file supp_RA117.000470_133922_0_supp_23978_fzffwf.zip › spectra_annotation/mito_spectra_annotation/468-1-4-1-5-1.pdf]

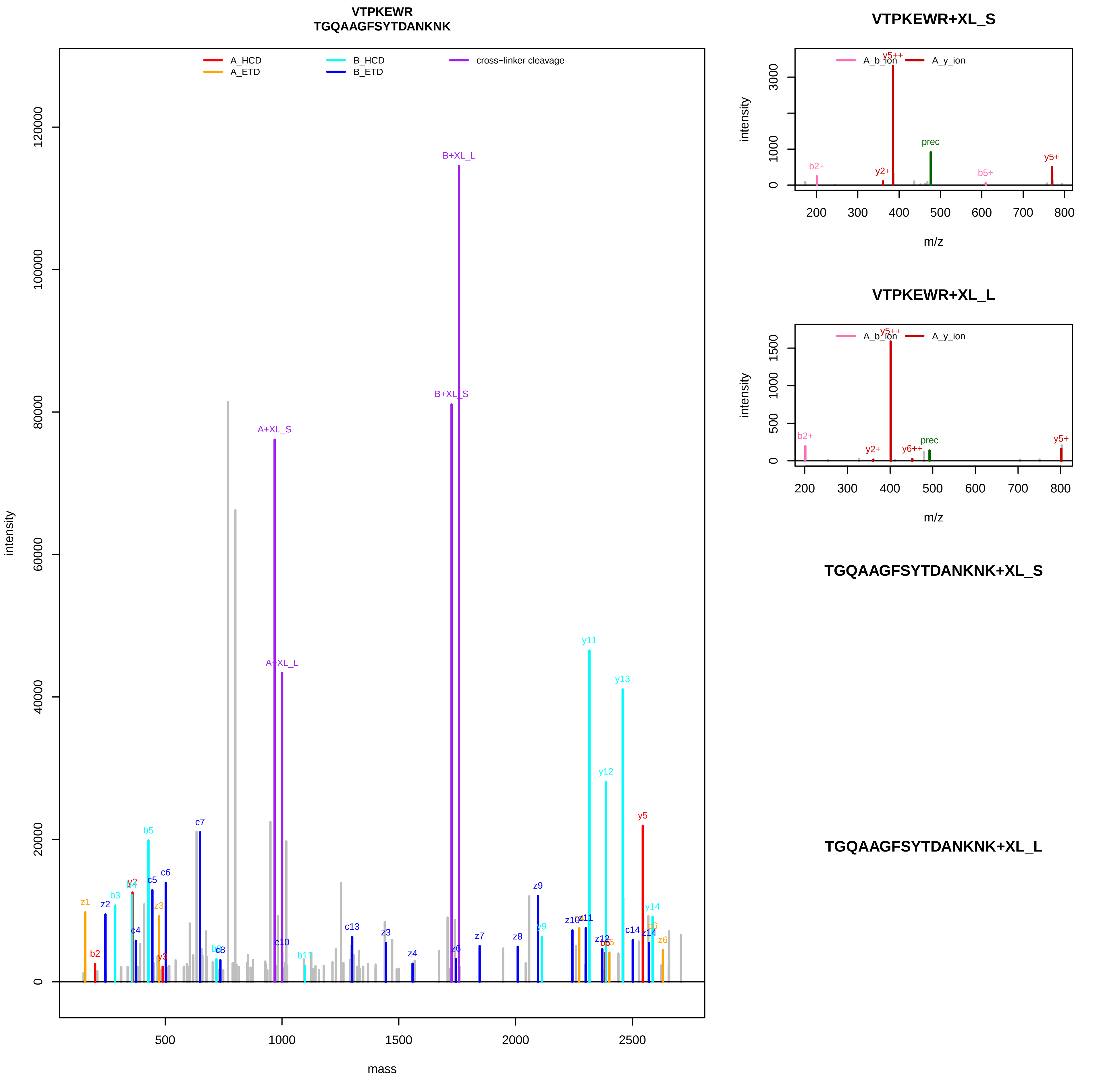
VTPKEWR
TGQAAGFSYTDANKNK
A_HCD
A_ETD
B_HCD
B_ETD
cross−linker cleavage
B+XL_L
B+XL_S
A+XL_S
A+XL_L
y11
y13
y12
y5
y14
z1
b2
z2
b3
b4
y2
c4
b5
c5
z3
y3
c6
c7
b6
c8
c10
b11
c13
z3
z4
z6
z7
z8
z9
y9
z10
z11
z12
b5
c14
z14
c6
z6
intensity
mass
0
20000
40000
60000
80000
100000
120000
500
1000
1500
2000
2500
VTPKEWR+XL_S
A_b_ion
A_y_ion
y5++
prec
b2+
y2+
b5+
y5+
intensity
m/z
0
1000
3000
200
300
400
500
600
700
800
VTPKEWR+XL_L
A_b_ion
A_y_ion
y5++
b2+
y2+
y6++
prec
y5+
intensity
m/z
0
500
1000
1500
200
300
400
500
600
700
800
TGQAAGFSYTDANKNK+XL_S
TGQAAGFSYTDANKNK+XL_L
